# Non-Melanoma Skin Cancer in Outdoor Workers: A Study on Actinic Keratosis in Italian Navy Personnel

**DOI:** 10.3390/ijerph17072321

**Published:** 2020-03-30

**Authors:** Luigi Vimercati, Luigi De Maria, Antonio Caputi, Enza Sabrina Silvana Cannone, Francesca Mansi, Domenica Cavone, Paolo Romita, Giuseppe Argenziano, Alessandro Di Stefani, Aurora Parodi, Ketty Peris, Massimiliano Scalvenzi, Giampiero Girolomoni, Caterina Foti

**Affiliations:** 1 Interdisciplinary Department of Medicine (DIM), Unit of Occupational Medicine, University of Bari, 70124 Bari, Italy; luigi.demaria@uniba.it (L.D.M.); antonio.caputi@uniba.it (A.C.); enza.cannone@uniba.it (E.S.S.C.); francesca.mansi@uniba.it (F.M.); domenica.cavone@uniba.it (D.C.); 2 Department of Biomedical Science and Human Oncology, Dermatological Clinic, University of Bari, 70124 Bari, Italy; romitapaolo@gmail.com (P.R.); caterina.foti@uniba.it (C.F.); 3 Dermatology Unit, Department of Mentals and Physical Health and Preventive medicine, University of Campania Luigi Vanvitelli Naples, 80138 Naples, Italy; 4 Institute of Dermatology, Università Cattolica, 00168 Rome, Italy; alessandro.distefani@policlinicogemelli.it (A.D.S.); ketty.peris@policlinicogemelli.it (K.P.); 5 Fondazione Policlinico Universitario A. Gemelli IRCCS, 00168 Rome, Italy; 6 Section of Dermatology, Di.S.Sal. Department of Health Science, University of Genoa, San Martino Polyclinic Hospital, 16132 Genoa, Italy; aurora.parodi@unige.it; 7 Dermatology Unit, Department of Clinical Medicine and Surgery, University of Naples Federico II, 80138 Naples, Italy; massimiliano.scalvenzi@unina.it; 8 Department of Medicine, Section of Dermatology, University of Verona, 37134 Verona, Italy; giampiero.girolomoni@univr.it

**Keywords:** ultraviolet exposure, non-melanoma skin cancer, actinic keratosis, Italian Navy

## Abstract

Occupational exposure to ultraviolet radiation is one of the main risk factors for non-melanoma skin cancer (NMSC) development. The most common variants of NMSC are basal cell carcinomas, squamous cell carcinomas, and actinic keratosis (AK). The latter is nowadays considered by most authors as an early squamous cell carcinoma rather than a precancerous lesion. Outdoor workers have a higher risk of developing NMSC because they spend most of the working day outside. The aim of this descriptive study was to assess the prevalence of skin lesions, especially AK, in a professional category of individuals exposed to ultraviolet (UV) radiation: the Italian Navy. From January to June 2016, a questionnaire and a total skin examination of 921 military personnel were administered by medical specialists (dermatologists) in seven different Italian Navy centres. AK was detected in 217 of 921 (23.5%) workers. Older age, outdoor occupation, longer working life, and fair skin seem to promote the development of AK. Of the 217 workers with AK, 187 (86.2%) had lesions in chronically sun-exposed skin areas. Italian Navy personnel have a high AK prevalence. Further studies are needed to investigate occupational hazards and their health effects among outdoor workers to promote protective behaviour and raise awareness of skin cancer.

## 1. Introduction

Non-melanoma skin cancer (NMSC) is the most widespread cancer in the world, with a tremendous individual and socioeconomic impact [1,2]. In Italy, the calculated incidence of NMSC is 119.4 cases for every 100,000 men and 90.7 cases for every 100,000 women per year [3]. The most common variants of NMSC are basal cell carcinomas (BCC), squamous cell carcinomas (SCC), and actinic keratosis (AK). AK, once considered a precancerous lesion, is nowadays considered an early squamous cell carcinoma in situ and is increasingly called keratinocyte intra-epidermal carcinoma [1,4]. These skin lesions are denominated as NMSC or “white skin cancer” to separate them from melanoma or “black skin cancer” [4]. SCC has a destructive development and a propensity to metastasize and usually derives from AK following a multistage model of keratinocyte cancer progression [5]. AK occurs mainly in chronically sun-exposed skin areas, such as the back of the hands, forearms, face, and scalp (especially in men), and may be accompanied by other signs of photodamage, such as telangiectasia, cutaneous dyschromia, solar elastosis, or skin atrophy [6].

It has been estimated that 50–70% of SCC and 50–90% of BCC in white individuals are caused by ultraviolet (UV) exposure [7]. In fact, although genetic factors and phenotype characteristics undoubtedly contribute to the development of skin cancer (e.g., light skin, light eyes, blonde or red hair, dysplastic nevi or many common nevi, skin burns, freckles, personal or family history of skin cancer) [8], the solar UV radiations represent the main cause of skin cancer [6,9,10].

Since 2012, the International Agency for Research on Cancer (IARC) and the World Health Organization (WHO) have classified ultraviolet radiation as “carcinogenic to humans” [11] for causing both malignant melanoma and NMSC. Other potential causes of skin cancer are arsenic, polycyclic aromatic hydrocarbons [12,13,14], and X-rays [15]. All these agents are also classified in “Group 1” by the IARC, and the Italian National Institute for Insurance against Accidents at Work (INAIL) remunerates cases of NMSC caused by these substances. Furthermore, theoretical models supported by recent studies suggest that other new potential factors (i.e., radon, air pollution) may contribute to risk of skin cancer [16,17,18,19]. Several authors found an association between professional sun exposure and the risk of developing NMSC [2,20,21,22,23]; furthermore, a higher rate of skin cancer (in particular, BCC and SCC) was detected in engineers, bricklayers, farmers, lifeguards, mountain guides, and mail carriers [2,4,23,24,25,26,27,28,29]. This relationship can arise mainly from prolonged and intense exposure to the sun and from the inappropriate use of sunscreen during working hours [10,28].

A category of workers particularly exposed to UV radiation during working hours is military personnel. In a recent review concerning active duty and veteran personnel, Riemenschneider et al. described a higher rate of cutaneous cancer among US soldiers compared to that among the general population. The same authors showed a low rate of skin neoplasm awareness and dermatological care among military personnel [30]. Among military corps, the Navy consists of workers performing different tasks with different occupational hazards, such as UV and asbestos hazard. In this population, asbestos is one of the most common and well-known causes of cancer, which is used in shipbuilding and is responsible for several types of cancer, in particular, pleural and peritoneal mesotheliomas [31]. This relationship is well established and is documented in the scientific literature; furthermore, the associated genetic mutations for asbestos-related diseases are also extensively documented [32,33,34,35,36]. In contrast, the number of scientific studies focusing on skin cancer in this professional category is very limited, although these individuals work in occupations with significant sun exposure [37]. 

The aim of this descriptive study was to assess the prevalence of skin lesions, particularly AK, in a professional category exposed to UV rays: the Italian Navy.

## 2. Materials and Methods

Data were selected from the “Love for the sea is in our skin” project, a national information and screening campaign promoted by the Italian Society of Professional and Environmental Allergological Dermatology (SIDAPA) and the Italian Society of Medical, Chirurgical, Esthetical Dermatology, and Sexually Transmitted Diseases (SIDeMaST) among Italian Navy personnel.

From January to June 2016, the cancer meetings and screening campaigns were performed in seven different Italian Navy centres:

- Taranto, Military Hospital—19 January 2016,
- Bari, Port Authority—16 February 2016,
- Brindisi, Barracks Carlotto—18 February 2016,
- Napoli, Naval Base—14 June 2016,
- La Spezia, District Bruno Falcomatà—15 June 2016,
- Roma, Barracks Grazioli Lante—15 June 2016,
- Augusta, District Terravecchia Marisicilia—23 June 2016,

After written informed consent was obtained from all participants, a questionnaire (available in the Supplementary Materials) and a total skin examination to detect skin lesions were administered by medical specialists (dermatologists). In case of suspicious lesions, the lesion was excised, and a histological examination was performed. Questionnaire items included demographic and clinical data (age, sex, personal history of skin cancer, indoor–outdoor main occupation, length of service, phenotype, clinical signs of facial skin ageing, AK lesions (number, type, and body site), and other suspected lesions (melanoma, SCC, BCC). None of the participants ever used sunscreen during work activities.

A preliminary univariate and multivariate statistical analysis of the data was performed using STATA 12 software (Statacorp, College Station, TX, USA).

## 3. Results

The overall sample consisted of 921 military personnel (average age of 40 years old). The adherence rate in the study was 100% (921 male military personnel). Table 1 shows the general characteristics of the study population.

### 3.1. Facial Skin Ageing

Solar lentigo or seborrheic keratosis were detected in 41.7% of individual examined. In addition, signs of sun damage such as cutaneous hyper- or hypopigmentation were observed in 39.6% of Navy personnel, while deep wrinkles or solar elastosis were identified in 25% of subjects. No signs of skin ageing were observed in 37.8% of cases.

Table 2 shows the prevalence of signs of facial skin ageing by age group and type of occupation (indoor, outdoor, indoor–outdoor). As expected, the prevalence increases with age and is higher among outdoor workers. In particular, we found that 55.4% of outdoor workers showed cutaneous hyper- or hypopigmentation versus 38.4% of indoor workers.

We also stratified the results by skin phototype (Fitzpatrick scale, Table 3). Workers with phototype I showed cutaneous hyper- or hypopigmentations (55.6%) and solar lentigo or seborrheic keratosis (55.3%). People with phototype IV and V, which were prevalent in 53% and 66.7% of the sample, respectively, showed no clinical sign of skin ageing.

### 3.2. Actinic Keratosis

AKs were present in 217 of 921 subjects (23.5%): 200 subjects (21.7%) showed less than five AK, and 17 subjects (1.8%) showed more than five AKs.

Table 4 shows the prevalence of AK by age group, type of occupation (indoor, outdoor, indoor–outdoor), and years of work.

Older age, outdoor occupation, and longer working life appeared to be associated with a higher prevalence of skin lesions. However, univariate and multivariate statistical analyses did not show statistically significant associations with age, main occupation, and years of work variables.

Data analysis also shows a significant odds ratio (OR) for AK adjusted for phototypes I and II (OR: 1.8; C.I. 95% (1.01–7.4)) (Table 5, Supplementary Materials Table S1).

Clinically, detected AKs were subcategorized according to the following characteristics [38]:

- “More palpable than visible” (type 1) in 85.4% of cases
- “Palpable and visible” (type 2) in 13.5% of cases
- “Hyperkeratotic lesions” (type 3) in 1.1% of cases

Type 3 of AK was reported in older subjects and in workers with a length of service beyond 31 years (Table 6).

Of the 217 workers with AK, 187 (86.2%) had lesions in chronically sun-exposed skin areas; in particular, 149 individuals (80.5%) showed AK on the face, 30 (16.2%) on the scalp, and 8 (4.3%) on the hands.

People over 60 years old with a light skin type (phototypes I–II) tended to develop AK on the scalp. Apart from skin type, the face was the most commonly affected area of the body (Table 7).

## 4. Discussion

The present study investigated skin lesions, particularly AK, in relation to natural UV occupational exposure in a large group of 921 Italian Navy personnel, one of the largest occupational groups studied in Italy. Despite this strength, our study has some limitations. First, a possible selection bias could be that the study population includes only subjects interested in the project “Love for the sea is in our skin” and consequently concerned about the health of their skin. Second, we lack data on subjects’ exposure to solar UV, although it can be assumed that all of them were exposed to a high level of solar UV during deployment off the coast of Italy in their whole Navy career [39]. Third, we will come to more reliable results if we can get information regarding the location they lived in during childhood.

The variability of UV irradiance is large throughout Italy due to the complex topography and large latitudinal extension of the country with an annual UV dose ratio between northern and southern Italy of about 1:2. The urban areas are generally characterized by low UV dose levels, due to higher aerosol optical depths, mostly of anthropogenic origin. Cities in northern Italy (industrialized areas) are characterized by lower doses than southern cities, particularly during the winter months (10–14 kJ m^-2^ day^-1^), due to less sun illumination and higher frequency of episodes of low horizontal visibility caused by pollution and fog [40,41].

Currently WHO/ILO are developing an evaluation of the global burden of melanoma and NMSC in solar UV-exposed workers [42], and several scientific studies on this issue are reported in the literature [1,10,21,23,43,44,45,46,47,48]. 

Interestingly, data collected from our study suggested that the overall prevalence of AK in Italian Navy personnel, a professional category of individuals often exposed to UV radiation, was higher than expected. Indeed, in this particular category of workers, we observed an AK prevalence of 23.5%; these data are significantly higher than those of the Prevalence of Actinic Keratoses Italian Study (PRAKTIS) study in the Italian general population, in which 1.4% of the subjects were affected by AK [49]. In addition, the result of our study is in line with that of a previous European population-based study that observed an AK prevalence of 23% [50]. In contrast, Flohil et al. obtained a different result in a Dutch population-based study that included more than 2000 individuals, in which almost 38% of participants had one or more AKs and 8% had 10 or more AKs. This high prevalence may be explained by the fact that compared with the prevailing characteristics of the Italian population (darker skin, hair, and eyes), the phenotypic characteristics of Dutch people (light skin, hair, and eyes) increase the risk for the appearance and development of AKs [51]. Our study confirmed the increased risk of AK observed in people with a light skin type: the highest number of AKs was found in subjects with Fitzpatrick phototype I (prevalence of 41.1%). Indeed, epidemiological data concerning the risk of AK confirmed a high prevalence in Caucasian people in both hemispheres [52].

Furthermore, we found a higher prevalence of skin lesions in the elderly population (AK prevalence of 36.3% among 60 year olds). This conclusion was confirmed by epidemiological evidence: for example, in the United Kingdom in the two-year period 2014–2016, an average of 47% of new NMSC cases per year were diagnosed in people aged 75 or over [53], and the AK prevalence in subjects over 70 years of age was 34% in men and 18% in women. With respect to the latter data regarding gender prevalence, Fartasch et al. [4] admitted that this difference between men and women was related to the higher sun exposure of men during outdoor work.

Our results showed a higher prevalence of the clinical signs of skin ageing (e.g., cutaneous dyschromia) and AK in outdoor workers (26.9% vs. 21.8% in indoor workers) who spent a longer period of their lives working. The IARC indicated that outdoor employees are exposed to a UV radiation dose that is two- to three-fold higher than that to which indoor workers are exposed [54], and several studies have confirmed a significant solar UV exposure during working time and higher skin cancer risk in outdoor workers [3,10,21,45,46,47,48,55]. Data collected in the EPIDERM outdoor worker project [56] (a European research project) concluded that outdoor employees had high-risk behaviour that exposed them more to the harmful effects of UV rays, despite having constitutional risk factors for developing NMSC comparable to those of indoor workers. These data were also confirmed by Trakatelli et al. [23], who, in a large European case-control study, showed that outdoor workers used less sunscreen and were more exposed to UV rays during work and free time. Similar results were recently confirmed also in a population attending Italian dermatology clinics, showing an odds ratio of 1.9 (1.4–2.4) in people working outdoors more than six hours/day [57]. These observations are in line with the detection of signs of significant photodamage in outdoor workers.

The scientific and medical literature shows consistent findings that AK is caused by cumulative sun exposure. Green [58] suggested that AK epidemiology reflects the following mechanism: skin lesions are generally localized in body areas mostly exposed to sunlight (head, neck, backs of hands, and forearms) in older, fair-skinned populations. The results of our study are consistent with these observations: older age, outdoor occupation, longer working life, and light skin type appeared to promote the development of skin lesions. In addition, of the 217 workers with AK, 187 (86.2%) had lesions in chronically photo-exposed skin areas; in particular, 149 individuals (80.5%) showed AK on the face. An interesting result was that people over 60 years old with a light skin type tended to develop AK on the scalp. This conclusion was probably associated with baldness, which frequently affects this age group.

The results of the analysis of the clinical subtypes of AK showed that severe subtypes of AK (type 3) were more frequent in the older study population (6.7% in subjects over 60 years old) and in workers with a length of service beyond 31 years (4.4%). Consistent with the findings of our study, a recent Italian review of occupational risk to sun exposure [59] concluded that outdoor work results in repetitive UV ray exposure, with possible cumulative photochemical damage in the skin of workers who, after many years, can experience damage, including AK, which may become progressively worse. In fact, in people continuously exposed to UV radiation, AKs can be multiple, and they can develop into hyperkeratotic lesions clustered in larger fields, [58], increasing the risk of progression into invasive SCC. 

## 5. Conclusions

The preliminary data of this study suggest that Italian Navy personnel, particularly outdoor workers, have a remarkable prevalence of NMSC. However, considering the lack of data on this category of employees, more work is needed to assess skin cancer risk. The majority of our study population had never before consulted a physician for a skin examination, which underlines the need to increase knowledge on UV exposure risk and protection behaviour among outdoor workers. Therefore, it seems necessary to launch awareness-raising campaigns to mitigate exposure to UV radiation by promoting workers’ education and use of personal protective equipment (e.g., use of covering clothes, hats, sunglasses, and sunblock; cessation of exposure in the mid-afternoon). Regarding personal protective equipment (PPE), the use of sunglasses should not be underestimated because solar UV is also a significant risk for eye health in workers exposed especially in high-reflectance environments, such as seafarers [60]. Secondary prevention includes health surveillance performed by occupational physicians and dermatologists [7,59,61].

Future studies should focus on knowledge about occupational hazards and subsequent health effects among outdoor workers, focusing in particular on Navy personnel, as well as promote protective behaviour and correct management, raising the awareness of skin cancer [62]. This approach may reduce the risk of work-related diseases, promote appropriate health education, and improve the quality of life of workers.

## Figures and Tables

**Table 1:** Demographic and clinical characteristics of the study population (n = 921).

Factors	%
Male	100%
Age	100%
40–50 years	66.4%
51–60 years	28.5%
≥60 years	5.1%
**Main occupation**	100%
Indoor	46.9%
Outdoor	26.3%
Indoor and outdoor	26.8%
**Length of service**	100%
1–10 years	5.7%
11–20 years	22.0%
21–30 years	48.7%
≥31 years	23.6%
**Phototype**	100%
I	4.3%
II	28.3%
III	51.4%
IV	15.5%
V	0.5%

**Table 2:** Signs of facial skin ageing by age and main occupation.

Signs	Age (%)			Main Occupation (%)		
	40–50	51–60	≥60	Indoor	Outdoor	Indoor–Outdoor
Solar lentigo or seborrheic keratosis	36.7	51.0	52.3	40.1	45.1	41.3
Cutaneous hyper- or hypopigmentation	36.9	43.4	50.0	38.4	55.4	25.7
Deep wrinkles or solar elastosis	36.7	51.0	52.3	40.1	45.1	41.3
No signs of facial skin ageing	43.1	28.9	20.5	39.9	26.6	45.7

The total is not 100% because more skin lesions were observed in the same subject.

**Table 3:** Signs of facial skin ageing by phototype (%).

Signs	Phototypes				
	I	II	III	IV	V
Solar lentigo or seborrheic keratosis	55.3	47.6	42.2	25.8	33.3
Cutaneous dyschromia	52.6	45.6	38.1	30.3	-
Deep wrinkles or solar elastosis	28.9	29.4	25.8	13.6	-
No signs of facial skin ageing	23.7	32.7	37.4	53.0	66.7

The total was not equal to 100, as multiple answers were possible.

**Table 4:** Prevalence of actinic keratosis (AK) by age group, type of occupation (indoor, outdoor, indoor–outdoor), and years of work.

Number of AKs	Age			Main Occupation			Years of Work			
	40–50	51–60	≥60	Indoor	Outdoor	Indoor–Outdoor	1–10	11–20	21–30	≥31
No AK	78.1	74.7	63.7	77.0	73.1	78.6	94.1	81.3	73.0	74.1
<5	20.9	22.6	27.7	21.8	26.9	16.6	5.9	18.7	25.4	22.2
5–10	0.8	2.3	4.3	0.7	-	4.0	-	-	1.4	2.8
>10	0.2	0.4	4.3	0.5	-	0.8	-	-	0.2	0.9
TOTAL	100	100	100	100	100	100	100	100	100	100

**Table 5:** Number of AKs for phototypes (%).

Number of AKs	Phototypes				
	I	II	III	IV	V
No AK	58.9	77.1	77.6	76.6	80.0
<5	35.9	19.8	21.1	22.7	20.0
5–10	2.6	2.3	1.1	0.7	-
>10	2.6	0.8	0.2	-	-
TOTAL	100	100	100	100	100

**Table 6:** Prevailing subtype of AK for age and years of service.

AK Type	Age			Years of Service			
	40–50	51–60	≥60	1–10	11–20	21–30	≥31
I	90.8	81.4	60.0	100	96.7	88.5	71.2
II	9.2	16.7	33.3	-	3.3	11.5	24.4
III	-	1.9	6.7	-	-	-	4.4
TOTAL	100	100	100	100	100	100	100

**Table 7:** Skin areas for age and phototype (%).

Skin Areas	Age			Phototypes				
	40–50	51–60	≥60	I	II	III	IV	V
Face	82.8	77.4	80.0	69.2	70.6	86.5	89.3	
Scalp	9.5	22.6	46.7	30.8	23.5	12.4	7.1	100.0
Hands	3.4	3.8	13.3	7.7	11.8	1.1	-	
Other body areas	12.1	9.4	20.0	15.4	7.8	13.5	14.3	

The total was not equal to 100, as multiple answers were possible.

## Supplementary Materials

The following are available online at https://www.mdpi.com/1660-4601/17/7/2321/s1, Table S1: Adjusted risks (ORs) for AKs by phototypes.
