# Supplementary material for: Non-Melanoma Skin Cancer in Outdoor Workers: A Study on Actinic Keratosis in Italian Navy Personnel
Source: Int J Environ Res Public Health. 2020 Mar 30;17(7):2321. doi: 10.3390/ijerph17072321 (PMC7177289; doi:10.3390/ijerph17072321)
Supplement: Supplementary file 1 [file ijerph-17-02321-s001.pdf]

Supplementary materials Table S1. Adjusted risks (ORs) for AKs by phototypes.

Variables	ORs	LCI (95%)	UCI (95%)
No AK	1.00	-	-
**Phototypes I,II**	1.81	1.01	7.40
**Phototypes III, IV, V**	0.42	0.21	12.73
